# A novel RT-QPCR-based assay for the relative quantification of residue specific m6A RNA methylation

**DOI:** 10.1038/s41598-019-40018-6

**Published:** 2019-03-12

**Authors:** Ainara Castellanos-Rubio, Izortze Santin, Ane Olazagoitia-Garmendia, Irati Romero-Garmendia, Amaia Jauregi-Miguel, Maria Legarda, Jose Ramon Bilbao

**Affiliations:** 1 Department of Genetics, Physical Anthropology and Animal Physiology, University of the Basque Country (UPV-EHU), Biocruces-Bizkaia Health Research Institute, Leioa, Spain; 2 IKERBASQUE, Basque Foundation for Science, Bilbao, Spain; 3 Department of Biochemistry and Molecular Biology, University of the Basque Country (UPV-EHU), Endocrinology and Diabetes Research Group, Biocruces-Bizkaia Health Research Institute, Leioa, Spain; 4 Pediatric Gastroenterology Unit, Cruces University Hospital, University of the Basque Country (UPV-EHU), Barakaldo, Spain; 5 Spanish Biomedical Research Center in Diabetes and Associated Metabolic Disorders (CIBERDEM), Madrid, Spain

## Abstract

N6-methyladenosine (m6A) is the most common and abundant RNA modification. Recent studies have shown its importance in the regulation of several biological processes, including the immune response, and different approaches have been developed in order to map and quantify m6A marks. However, site specific detection of m6A methylation has been technically challenging, and existing protocols are long and tedious and often involve next-generation sequencing. Here, we describe a simple RT-QPCR based approach for the relative quantification of candidate m6A regions that takes advantage of the diminished capacity of *BstI* enzyme to retrotranscribe m6A residues. Using this technique, we have been able to confirm the recently described m6A methylation in the 3′UTR of *SOCS1* and *SOCS3* transcripts. Moreover, using the method presented here, we have also observed alterations in the relative levels of m6A in specific motifs of SOCS genes in celiac disease patients and in pancreatic β-cells exposed to inflammatory stimuli.

## Introduction

N6-methyladenosine (m6A) is the most abundant internal modification on mammalian mRNAs and long-noncoding RNAs. These modifications are generally dynamic and exhibit changes in distribution or frequency in response to certain stimuli, constituting a new layer of gene regulation termed epitranscriptomics^1,2^. Genetic loss-of-function studies for m6A methyltransferases (m6A writers), m6A-binding proteins (m6A readers), and m6A demethylases (m6A erasers) have highlighted a critical role for m6A modifications in the control of gene expression in a range of physiological processes^3,4^. A recent study analyzing the effects of deleting writer *Mettl3* in mouse T-cells found that, in the absence of *Mettl3*, mice develop a chronic inflammation of the intestine and that the mRNAs for STAT signaling inhibitory proteins SOCS1 and SOCS3, that generally present m6A methylation marks in their 3′UTR region, exhibited slower decay, resulting in increased mRNA and protein levels, and establishing a link between the m6A methylation of those transcripts and T-cell responses^5,6^. More recently, m6A has been shown to affect the LPS-induced inflammatory response in human dental pulp cells^7^ opening a new layer of investigation into the molecular mechanisms of inflammatory diseases.

Technological advances in high-throughput sequencing in combination with antibody-enrichment and/or induced nucleotide-specific chemical modifications have accelerated the mapping of epitranscriptomic modifications. However, site-specific detection and quantification of m6A has been technically challenging^8^. Methods developed so far involve long and tedious procedures like SCARLET (site-specific cleavage and radioactive-labeling followed by ligation-assisted extraction and thin-layer chromatography)^9^ and miCLIP (m6A individual-nucleotide-resolution cross-linking and immunoprecipitation)^10^ or are based on the diminished capacity of different nucleic acid polymerases, namely *Tth (Thermus thermophiles)* and *BstI (Bacillus stearothermophilus)*, to retrotranscribe m6A nucleotides located adjacent to the priming oligo^11,12^. Flexibility is necessary for the processive movement of the enzyme during retrotranscription and the conformational freedom between the complex formed by the primer, the template and the enzyme itself is decreased in RNA templates that contain m6A residues^12,13^. Single-nucleotide primer extension with these enzymes followed by polyacrylamide gel electrophoresis of the extension products has already been used to quantify the m6A methylation levels of specific sites^11,12,14^. In the present study, we have simplified the protocol while improving accuracy in the relative quantification of m6A nucleotides by taking advantage of the inherent reverse transcriptase activity of *BstI* polymerase, hampered in the presence of m6A as previously described by Shi, C *et al*.^15^ in combination with relative quantification using PCR. We have used this novel m6A-RT-QPCR approach to quantify the m6A levels in specific residues of human *SOCS1* and *SOCS3* mRNAs, members of one of the major mechanisms for the regulation of cytokine signaling^16^, in intestinal and β-pancreatic cell lines and in intestinal biopsy samples. Thus, the aim of this study was to develop a simple and cost-effective method to relatively quantify the m6A levels of candidate transcripts that could contribute to the development of different types of diseases, such as those related to the immune system.

## Results

### m6A-induced reduction in *BstI* retrotranscription efficiency can be assessed by QPCR

**Figure 1:**
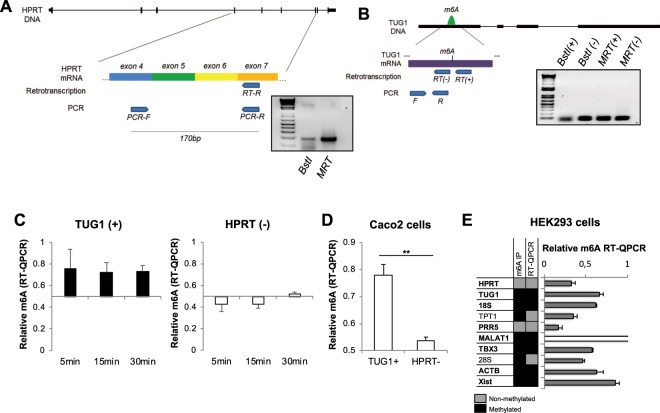
(**A**) Graphical representation of retrotranscription followed by PCR of *HPRT* (left). Agarose gel after PCR of the retrotranscribed samples using *BstI* and *MRT* enzymes (right). (**B)** Graphical representation of retrotranscription followed by PCR of *TUG1* lncRNA. m6A mark (in green) was described by SCARLET method (Liu, N. *et al*.^9^) (left). Agarose gel after PCR of the retrotranscribed samples using m6A (+) and (−) primers and *BstI* and *MRT* enzymes. (**C**) m6A-RT-QPCR using different time points (5 min, 15 min and 30 min) for the retrotranscription of methylated *TUG1* and unmethylated *HPRT* mRNAs. RNA was extracted from three different passages of HCT15 intestinal cell line. (**D**) Relative quantification of m6A levels of *TUG1* and *HPRT* in Caco 2 intestinal cells. Data are represented as the mean and standard error of three independent experiments. **p-value < 0.01 based on paired Student’s t-test. (**E**) Comparison of m6A IP data retrieved from the Met-DB v2.0 database and relative m6A RT-QPCR results in different genes using the HEK293 cell line. Genes in bold showed concordant results using both methods. Grey boxes represent negative (non-methylated) and black boxes represent positive (methylated) results. Relative RT-QPCR data correspond to the mean and standard error of four independent experiments.

It has been previously shown that *BstI* polymerase has an inherent retrotranscriptase (RT) activity^12,15^, but we wanted to evaluate its capacity to retrotranscribe mRNA using dNTPs instead of a single nucleotide. We designed a reverse primer in exon 7 of *HPRT* gene and performed RT reactions using *BstI* or *M-MuLV* RT *(MRT)* enzymes and RNA extracted from HCT15 intestinal cells (Fig. 1A). To verify the ability of *BstI* for retrotranscription, we subsequently amplified the retrotranscription reaction products by PCR using a forward primer in exon 4 and a reverse primer in exon 7, which ensures that the product will be derived from mRNA and not from any trace of genomic DNA. Both enzymes are able to synthesize cDNA from an mRNA template but, as expected, *MRT* outperforms *BstI* in retrotranscription efficiency (Fig. 1A).

Having confirmed the retrotranscriptase activity of *BstI* polymerase in our experimental conditions, we wanted to evaluate its ability to reverse transcribe a known m6A residue in the *TUG1* lncRNA^7^. We performed two RT reactions; one with a primer located adjacent to the test m6A residue, or *primer* (+), and another one with no m6A nearby, or *primer* (−) (Fig. 1B). After PCR amplification of the RT product, we could detect differences in band intensities between the two reactions performed with *BstI*, suggesting a higher RT efficiency for the reaction in which *primer* (−) was used (Fig. 1B). On the contrary, PCR of the RT products from the *MRT* reactions suggest that this enzyme is not affected by the proximity of an m6A mark to the primer. In view of these results, we hypothesized that those differences in RT efficiency caused by the presence of m6A could be relatively quantified by a QPCR step following retrotranscription. In order to test this possibility, *HPRT* gene was used as a negative control for m6A (no methylation marks are described in this transcript), while *TUG1* was used as a positive control. The following formula was stablished for the calculation of the relative m6A levels after QPCR:

$$Relative\,m6{\rm{A}}={2}^{-(Ctprimer(-)BstI-Ctprimer(-)MRT)/(Ctprimer(+)BstI-Ctprimer(+)MRT)}$$

Based on these calculations, in the absence of m6A marks, the differences of retrotranscription efficiencies between the two enzymes will be similar regardless of the primer used, and the values of the relative m6A quantification will be close to 0.5. In the presence of an m6A residue, due to the decreased retrotranscription ability of *BstI*, the difference in RT efficiencies between enzymes will be more pronounced when using the positive (+) primer (adjacent to the m6A residue), resulting in higher values (>0.5) of relative m6A quantification.

In order to determine the best time-point for the quantification of m6A based on the differences in enzyme performance, we set up a m6A-RT-QPCR time-course for *TUG1* and *HPRT* genes in RNA samples extracted from three different passages of the human HCT15 intestinal cell line. As seen in Fig. 1C, the m6A-positive region in *TUG1* mRNA showed positive values (above 0.5) in all three time points. However, the m6A-negative region in the *HPRT* gene started to show values above the threshold after 30 minutes of retrotranscription, so all subsequent experiments were done after 15 minutes of retrotranscription. Under these conditions, m6A-RT-QPCR analyses confirmed the positive and negative m6A status in *TUG1* and *HPRT* genes, respectively, when using RNA extracted from Caco2 intestinal cell line (Fig. 1D), indicating that this method is useful to relatively quantify the m6A methylation at a single nucleotide level. To extend the applicability of the method, we quantified relative m6A methylation on several additional RNA residues and compared the results with previously published m6A immunoprecipitation data of the same regions available at the Met-DB v2.0 database^17^. For this purpose, we used RNA from HEK293 cells as it was the material used in the immunoprecipitation experiments. Eight out of the ten gene regions analyzed by relative m6A RT-QPCR method presented concordant results in terms of methylation status (methylated/non-methylated) when compared with those available in the database (Fig. 1E) confirming the high efficiency of this method.

### Fluctuations in m6A levels can be detected using m6A-RT-QPCR

**Figure 2:**
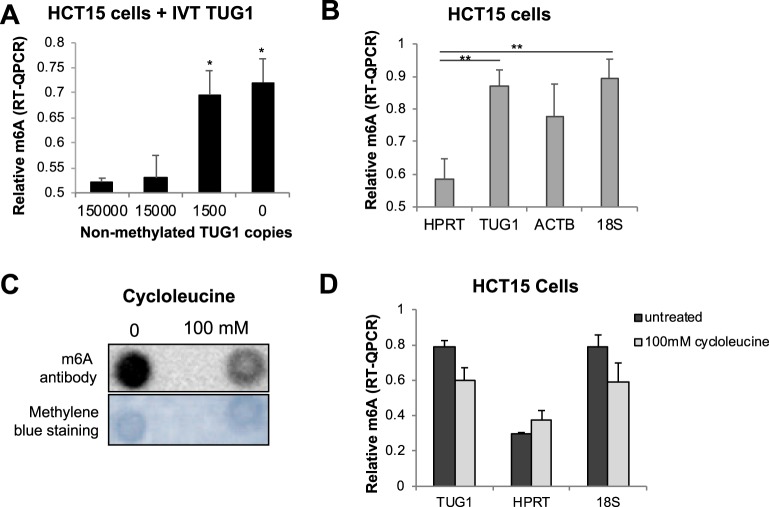
(**A**) Relative m6A quantification of *TUG1* motif in basal RNA (0) and in RNA mixed with increasing amount of copies (1500, 150000 and 1500000) of *in vitro* transcribed non-methylated RNA.   *p-value < 0.05 based on paired Student’s t-test. (**B**) Relative quantification of previously described methylated motifs using m6A-RT-QPCR method. **p-value < 0.01 based on paired Student’s t-test. (**C**) HCT15 cells were treated with different concentrations of cycloleucine 3 hours prior to RNA extraction and total RNA was evaluated for the quantification of the m6A signal. Representative m6A RNA immuno-dot blot of basal and 100 mM cycloleucine treatment of 4 independent experiments. Methylene blue staining was used as a loading control for RNA amount. (**D)** Relative quantification of the m6A levels in the selected motifs of *TUG1* and *18S* in RNA extracted from non-treated (−) and 100 mM cycloleucine treated cells. HPRT was used as a negative control. Data are represented as the mean and standard error of four independent experiments.

In order to assess the ability of this technique to detect fluctuations in m6A levels, the same *TUG1* region was *in vitro* transcribed (IVT) to generate a single stranded RNA without any methylation. Increasing amounts of non-methylated IVT RNA were mixed with RNA extracted from HCT15 cells and the ability to detect m6A marks was quantified. The methylation levels of *TUG1* residue decreased but remained positive after adding 1500 copies of non-methylated transcript and got closer to negative values after adding 150,000 and 1,500,000 copies of the non-methylated RNA (Fig. 2A).

To further confirm the suitability of this method for relative quantification of m6A levels in different RNA transcripts in the HCT15 cell line, we used primers adjacent to other described m6A motifs in *ACTB* mRNA and *18S* RNA^9,12^. As was the case for *TUG1*, both transcripts showed methylation in the selected motifs, however, only the levels observed in *18S* were statistically significant compared to the non-methylated control *HPRT* (Fig. 2B). To determine the ability of this method to quantify m6A fluctuation levels we incubated cells with different concentrations of cycloleucine, an inhibitor of m6A methylation^18^, in order to get RNA with decreased methylation percentage. Incubation of cells with 100 mM cycloleucine shows 35% average reduction in the general m6A signal detected by RNA Dot-Blot (Fig. 2C) using an anti-m6A antibody. These results were concordant with the relative quantification by m6A-RT-QPCR of the *TUG1* and *18S* regions, which showed a decreased abundance of m6A of 25% after cycloleucine treatment (Fig. 2D).

### Methylation in *SOCS1* and *SOCS3* 3′UTR can be assessed by m6A-RT-QPCR

**Figure 3:**
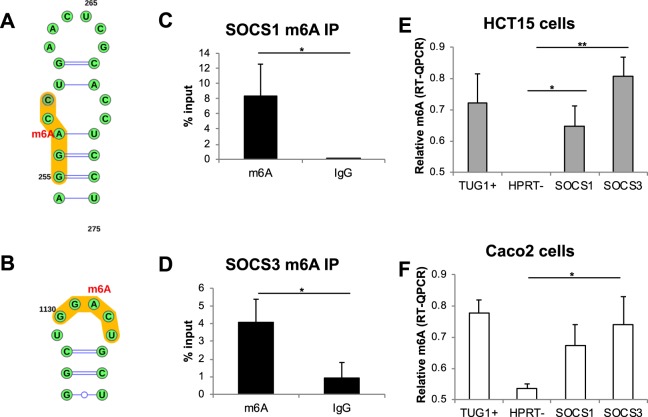
Location of the m6A motifs in the 3′ sites of the (**A**) *SOCS1* and (**B**) *SOCS3* genes. RNA immunoprecipitation of m6A followed by RT-QPCR for the 3′ sites of (**C**) *SOCS1* and (**D**) *SOCS3*. Data are represented as the mean and standard error of four independent experiments. p-value *p < 0.05. Validation of the m6A-RT-QPCR method for the relative quantification of m6A motifs in *SOCS1* and *SOCS3* genes in RNA extracted from (**E**) HCT15 and (**F)** Caco2 cells. Data are represented as the mean and standard error of three independent experiments. **p-value < 0.01, *p < 0.05; based on paired Student’s t-test.

In order to further validate our method for the quantification of m6A levels in additional transcripts, we analyzed the methylation levels of *SOCS1* and *SOCS3* mRNAs. The methylation of these two transcripts has been recently associated with their stability and subsequently with the ability of T-cells to respond to certain stimuli as the proinflammatory cytokine IL7^5^. As m6A marks in these genes have been described to be located in the 3′UTR, we analyzed their sequences in order to find the DRACH m6A consensus motif^10^. *SOCS1* presents a single consensus motif (GGACC), m6A-256 (Fig. 3A), while several motifs are found in the *SOCS3* gene. For the latter, we selected to evaluate one of the two GGACU motifs, m6A-1132, as it has been described to be the most commonly methylated sequence (Fig. 3B). We were able to confirm the methylation of the selected regions in both *SOCS1* and *SOCS3* in RNA from intestinal origin using RNA immunoprecipitation with an antibody against m6A followed by QPCR (Fig. 3C,D).

We next evaluated the mRNA methylation of *SOCS1* and *SOCS3* m6A motifs in Caco2 and HCT15 intestinal cell lines using our novel m6A-RT-QPCR method. Both intestinal cell lines showed significantly positive levels of m6A methylation in the *SOCS3* motif (Fig. 3E,F). The methylation levels of *SOCS1* reached statistical significance only in the HCT15 cells. These results confirm that this method can be used to relatively quantify the m6A methylation level of a single base in different transcripts.

### m6A-RT-QPCR detects inflammation related m6A methylation changes

To assess the ability of the m6A-RT-QPCR method to observe methylation changes, we evaluated the alterations in m6A methylation of the selected *SOCS1* and *SOCS3* motifs in different inflammation related scenarios. The mRNA methylation of these genes has been shown to be stimulus-specific in T-cells, where stimulation with IL7 induces m6A methylation and subsequent degradation of these transcripts^5^.

**Figure 4:**
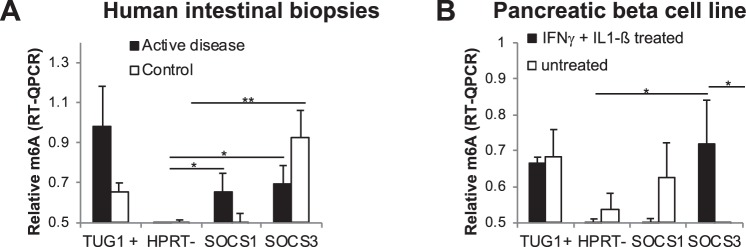
(**A**) Relative m6A quantification of selected *SOCS1* and *SOCS3* motifs in intestinal biopsies of celiac patients (n = 8) and controls (n = 6). Data are represented as the mean and standard error of all samples in each group. **p-value < 0.01; *<0.05 based on unpaired Student’s t-test. (**B**) Relative m6A quantification of selected *SOCS1* and *SOCS3* motifs in pancreatic β-cells stimulated with IFNγ and IL1β or without stimulation. Data are represented as the mean and standard error of three independent experiments. *p-value < 0.05 based on paired Student’s t-test.

Celiac disease (CeD) and type 1 diabetes (T1D) are chronic inflammatory conditions of the small intestine and the pancreas, respectively. SOCS genes have been involved in the pathogenesis of these diseases, and it is known that celiac and diabetic patients show alterations in these genes at both mRNA and protein levels^19,20^. We used RNA extracted from intestinal biopsy samples of celiac disease patients and non-celiac controls to analyze the levels of m6A methylation in the selected motifs of SOCS genes. Similarly to what happened in the intestinal cell lines, the *SOCS3* motif analyzed presented significantly higher m6A levels compared to *HPRT*. However, *SOCS1* levels were only significant in the active disease biopsies (Fig. 4A). The mean methylation of both motifs shows a change in the disease status, with a tendency to higher methylation levels for *SOCS1* and lower methylation for *SOCS3* motif in the celiac patient group (Fig. 4A). The m6A-RT-QPCR technique was also used to quantify the levels of m6A SOCS gene motifs in *in vitro* cultured pancreatic β-cells. In this case, cells were stimulated with IFNγ and IL1β to simulate the inflammatory environment characteristic of the β-cell destruction that occurs in T1D. In contrast to what we had observed in the intestinal biopsies, ß-cells treated with the pro-inflammatory cytokines showed no methylation of the *SOCS1* motif and a significantly higher methylation of the *SOCS3* motif (Fig. 4B).

## Discussion

N6-Methyladenine (m6A) is the most abundant internal modification on mammalian RNAs constituting a new layer of gene regulation that is involved in different biological processes as T-cell response or tumorigenesis^5,21^. Moreover, it has been shown that this modification plays important roles in the development of several disorders, as neuronal disorders, immune-mediated diseases, obesity and cancer^2^. Although several techniques have been developed in order to map and quantify m6A residues^9,10,22^, available methods are laborious or involve next-generation sequencing, so new approaches are needed to measure the m6A levels in selected transcripts that could be involved in the development of disease.

In this study we have taken advantage of the previously described diminished capacity of *BstI* enzyme to retrotranscribe m6A nucleotides located adjacent to the priming oligo^11,12^. Using the method described here we have been able to confirm the methylation motifs described by other techniques in *TUG1* lncRNA, *ACTB* mRNA and *18S* rRNA in intestinal cell lines, in a cost-affordable and time-efficient way. Additionally, when comparing the results of specific residues using our method to those of m6A immunoprecipitation experiments, where a region is evaluated, we found that the methylation status in concordant in eight out of ten of the RNAs further supporting the validity of the method.

This study shows that this method is useful for the relative quantification of a specific residue within a transcript. When a non-methylated RNA is added to basal RNA, decreasing the percentage of m6A, or cycloleucine is used to reduce the overall RNA methylation levels in the cell, values obtained using m6A-RT-QPCR decreased, confirming the validity of the technique to evaluate if a certain residue presents alterations in its methylation rates in response to different stimuli or disease status. Although the experiments performed using IVT RNAs suggests that m6A percentages as low as 1%, resembling what has been described for SCARLET^9^, could be detected using this technique, the exact percentages are not possible to determine due to the extreme difficulty to artificially synthesize a stable RNA with the same characteristics as the regions that are being analyzed (i.e 140 bp).

This technique can be used with limited amounts of RNA extracted from human tissues, that normally are not enough to perform  an RNA immunoprecipitation, and the relative amount of m6A marks in candidate regions can be easily quantified. We have demonstrated the usefulness of this approach for the quantification of residue specific methylation in the 3′UTR of *SOCS1* and *SOCS3* transcripts that were recently described to be regulated by m6A modifications in the context of immune response^5^. Moreover, using this technique, we have shown that there are inflammation-related changes in the mRNA methylation of specific motifs of these two genes. It has been known for a long time that SOCS proteins play an important part in the fine tuning of the immune response and inflammation^16^ being involved in inflammatory diseases as CeD or T1D, the inflammation-related variability of the m6A methylation in these transcripts, and other interesting candidates, could provide progress on the understanding on their contribution in disease pathogenesis.

## Material and Methods

### Biopsies and cell lines

Celiac disease in pediatric patients was diagnosed according to the ESPGHAN (European Society of Pediatric Gastroenterology Hematology and Nutrition) criteria in force at the time of recruitment, including anti-gliadin (AGA), antiendomysium (EMA) and anti-transglutaminase antibody (TGA) determinations as well as a confirmatory small bowel biopsy. The study was approved by the Cruces University Hospital Ethics Board and analyses were performed after informed consent was obtained from all subjects or their parents. All experiments were performed in accordance with relevant guidelines and regulations. Biopsy specimens from the distal duodenum of each patient were obtained during routine diagnosis endoscopy. None of the patients suffered from any other concomitant immunological disease. None of the controls showed small intestinal inflammation at the time of the biopsy.

Intestinal Caco2 (86010202) and HCT15 (91030712) cell lines were purchased from Sigma Aldrich. HCT15 cells were incubated with 100 mM cycloleucine (Sigma Aldrich) for 3 hours prior to RNA extraction. The EndoC-βH1 human β-cell line (provided by Dr. R. Scharfmann, Centre de Recherche de l’Institut du Cerveau et de la Moelle Épinière, Paris, France) was cultured in plates coated with Matrigel-fibronectin (100 mg/ml and 2 mg/mL, respectively) in low-glucose DMEM as previously described^23^. Human pancreatic ®-cells were exposed to IL-1ß (R&D Systems, Abingdon,U.K.) at 50 units/mL and IFNγ (PeproTech, Rocky Hill, NJ) at 1,000 units/mL for 24 h.

### RNA, retrotranscription, PCR and QPCR

Total RNA was extracted from cells and biopsies using QIAGEN RNA mini kit. All samples were subjected to DNAse I treatment during extraction.

For the m6A-retrotranscription reaction, 75–150 ng of RNA, 100 nM of each primer, 50 ⌠M dNTPs and 0.1U of *BstI* (NEB) or 0.8U of* MRT* (ThermoScientific) were used. The cycling conditions were as follows: 50 °C -5/15/30 min, 85 °C-3 min, 4°-∞. For the PCR, 1 ⌠l of the RT reaction was amplified using Dream Taq polymerase (ThermoScientific) and 100 nM of each primer. Cycling conditions were as follows: 95 °C-3min, 40 cycles x (95 °C-30sg, 60 °C- 30sg, 72 °C-30sg), 4 °C-∞. For the QCPR, 1.5 ⌠l of the retrotranscription reaction was used together with 100 nM of each primer and 2X iTaq SYBR green (BioRad). Reactions were run in an Illumina Eco Real Time System and melting curves were analyzed to ensure the amplification of a single product. The sequences of all the primers are available upon request.

### *In vitro* transcription (IVT)

*TUG1* amplicon was reamplified using a forward primer with a T7 sequence. After purification, 100 ng of DNA were used for *in vitro* transcription using T7 polymerase and rNTPs from Takara (Clonthech) following manufacturer’s instructions. The IVT product was purified using the Direct-zol miniprep kit (Zymo Research).

### Dot blot

For cycloleucine experiments 400 ng of RNA were crosslinked into a nitrocellulose membrane using UV. Membrane was blocked using 5% milk in 0.1% PBST (0.1% Tween in PBS). Membrane was incubated overnight with rabbit m6A antibody (1:500) (Abcam, ab151230) at 4 °C followed by an incubation with a secondary HRP anti-rabbit antibody. Membrane was developed using SuperSignal West Femto substrate (Thermofisher) in a ChemiDoc gel imaging system (Biorad).

### m6A RNA immunoprecipitation

2 μg of RNA per sample were fragmented with RNA fragmentation buffer (100 mM Tris, 2 mM MgCl_2_) for 3 min at 95 °C and placed on ice immediately after heating. 10% of RNA was kept as input. 1 µg of m6A antibody (Abcam, ab151230) was coupled to agarose A beads (GE Healthcare) in a rotation wheel for 1 h at 4 °C. After incubation, beads were washed twice in reaction buffer (150 mM NaCl, 10 mM Tris-HCl, 0.1% NP-40), RNA was added to the antibody coupled beads and incubated for 3 h at 4 °C in a rotating wheel. Subsequently, beads were washed 2X in reaction buffer, 2X in low salt buffer (50 mM NaCl, 10 mM TrisHCl and 0.1% NP-40) and 2X in high salt buffer (500 mM NaCl, 10 mM TrisHCl and 0.1% NP-40). After the last wash, beads were resuspended in Lysis buffer and RNA was extracted using the PureLink RNA extraction kit (Ambion).

### Statistical analysis

All experiments were performed in triplicate. The data are represented as the mean ± standard error of the mean. Comparisons were analyzed by Student’s t test. The significance level was set at p < 0.05.
